# Telehealth Behavioral Intervention for Diabetes Management in Adults With Physical Disabilities: Intervention Fidelity Protocol for a Randomized Controlled Trial

**DOI:** 10.2196/31695

**Published:** 2021-09-10

**Authors:** Ayse Zengul, Eric Evans, Allyson Hall, Haiyan Qu, Amanda Willig, Andrea Cherrington, Mohanraj Thirumalai

**Affiliations:** 1 Department of Nutrition Sciences University of Alabama at Birmingham Birmingham, AL United States; 2 Department of Health Services Administration University of Alabama at Birmingham Birmingham, AL United States; 3 Department of Medicine University of Alabama at Birmingham Birmingham, AL United States

**Keywords:** telehealth, health coaching, artificial intelligence, diabetes mellitus, intervention fidelity, mobile phone

## Abstract

**Background:**

Diabetes mellitus is a major health problem among people with physical disabilities. Health coaching has been proven to be an effective approach in terms of behavioral changes, patient self-efficacy, adherence to treatment, health service use, and health outcomes. Telehealth systems combined with health coaching have the potential to improve the quality of health care by increasing access to services. Treatment fidelity is particularly important for behavior change studies; however, fidelity protocols are inadequately administered and reported in the literature.

**Objective:**

The aim of this study is to outline all the intervention fidelity strategies and procedures of a telecoaching intervention—artificial intelligence for diabetes management (AI4DM)—which is a randomized controlled trial to evaluate the feasibility, acceptability, and preliminary efficacy of a telehealth platform in adults with type 2 diabetes and permanent impaired mobility. AI4DM aims to create a web-based disability-inclusive diabetes self-management program. We selected the National Institutes of Health Behavior Change Consortium (NIH BCC) fidelity framework to describe strategies to ensure intervention fidelity in our research.

**Methods:**

We have developed fidelity strategies based on the five fidelity domains outlined by the NIH BCC—focusing on study design, provider training, treatment delivery, treatment receipt, and enactment of treatment skills. The design of the study is grounded in the social cognitive theory and is intended to ensure that both arms would receive the same amount of attention from the intervention. All providers will receive standardized training to deliver consistent health coaching to the participants. The intervention will be delivered through various controlling and monitoring strategies to reduce differences within and between treatment groups. The content and structure of the study are delivered to ensure comprehension and participation among individuals with low health literacy. By constantly reviewing and monitoring participant progress and protocol adherence, we intend to ensure that participants use cognitive and behavioral skills in real-world settings to engage in health behavior.

**Results:**

Enrollment for AI4DM will begin in October 2021 and end in October 2022. The results of this study will be reported in late 2022.

**Conclusions:**

Developing and using fidelity protocols in behavior change studies is essential to ensure the internal and external validity of interventions. This study incorporates NIH BCC recommendations into an artificial intelligence embedded telecoaching platform for diabetes management designed for people with physical disabilities. The developed fidelity protocol can provide guidance for other researchers conducting telehealth interventions within behavioral health settings to present more consistent and reproducible research.

**Trial Registration:**

ClinicalTrials.gov NCT04927377; http://clinicaltrials.gov/ct2/show/NCT04927377.

**International Registered Report Identifier (IRRID):**

PRR1-10.2196/31695

## Introduction

### Background

Although diabetes mellitus (DM) is a significant problem among all populations, people with disabilities are more likely to be affected by DM. According to the 2017 Behavioral Risk Factor Surveillance System report, 23.6% of people with disabilities are diagnosed with diabetes, whereas 9% of people without a disability are diagnosed with diabetes [1]. DM can increase the overall risk of premature death and is linked to a number of complications, including heart attack, stroke, kidney failure, leg amputation, vision loss, and nerve damage [2]. Current science has enabled multiple approaches to manage type 2 diabetes (T2D) and its related complications [2-5]. Glycemic control through a combination of diet, physical activity (PA), medication adherence, and glucose monitoring have been shown to reduce glycated hemoglobin and be effective in T2D management for both the general population and people with intellectual and developmental disabilities [2,4,6,7].

Behavioral weight loss programs, such as the lifestyle interventions used in the Diabetes Prevention Program, have proven successful in DM management [8]. However, there is no accessible, inclusive, and adapted diabetes management program for people with disabilities. Programs that are not designed with people with disabilities in mind (ie, noninclusive) pose various physical, programmatic, and attitudinal barriers. To address various barriers, such as lack of time and transportation, and to reach millions of more people, several studies have successfully used technology-mediated interventions for DM management [4,6,9-11]. However, these technology-mediated intervention solutions are not inclusive and usually have static content delivered through websites, emails, or mobile apps with no personalized interaction with the client.

To address these deficiencies, we are developing a platform that combines human synchronous telecoaching with mobile health (mHealth) technologies to promote T2D self-management for people with disabilities. In this artificial intelligence (AI)–assisted, individualized, family-focused, lifestyle modification telehealth intervention (AI for DM [AI4DM]), we use an AI-embedded telecoaching dashboard to promote the fidelity of coaching sessions and reduce the workload required to personalize telecoaching.

### Importance of Reporting Fidelity

Fidelity in health behavior change studies is an essential factor in ensuring that the intervention program is delivered as intended [12]. Telehealth behavior change studies usually include similar strategies to deliver the treatment, such as using guidelines or manuals, monitoring audio and video materials, and adherence to the intervention protocols. However, several systematic reviews of health behavior interventions indicate that there is inconsistency in the use of these strategies. In addition, most of these interventions did not incorporate effective fidelity protocols to monitor the validity of their research [12-16]. To report their fidelity, studies usually focus on monitoring intervention delivery but poorly report and discuss other fidelity components, such as design, training, and intervention receipt strategies to enhance intervention fidelity [17]. Inadequate reporting of fidelity can lead to misinterpretation of the results because of the increased type 1 and type 2 errors due to the residual confounding bias, which would contribute to difficulty replicating and translating findings of the study into practice [12].

The complexity of research designs, working with diverse populations, and maintaining credibility when testing the feasibility of innovative interventions are common challenges faced by behavior change studies. The methodological challenges in designing, conducting, and reporting health behavior change studies eventually led to the formation of the Treatment Fidelity Workgroup of the National Institutes of Health Behavior Change Consortium (NIH BCC) [14]. In 2004, NIH BCC published recommendations to guide and encourage researchers to incorporate treatment fidelity concepts and strategies in the field of health behavior change. The recommendations focus on five domains: study design, provider training, delivery of treatment, receipt of treatment, and enactment of treatment. These recommendations include advancing the definition, methodology, and measurement of treatment fidelity to enhance the internal and external validity of the interventions.

Considering the importance of developing and using treatment fidelity in behavioral change interventions, we aim to describe and report the fidelity protocol for the AI4DM study. AI4DM combines mHealth technologies with telecoaching sessions to create the first-ever web-based diabetes self-management program for people with disabilities. The fidelity protocol was developed to address the five domains of intervention fidelity outlined by the NIH BCC. Considering the complexity of our intervention design, the development and use of the fidelity protocol is essential to document our fidelity measures to present more consistent and reproducible research.

## Methods

### Overview of the Study and the Intervention Fidelity Protocol

This project aims to develop and assess a telehealth framework, AI4DM, paired with inclusive diabetes self-management content. AI4DM is grounded in the social cognitive theory (SCT) [18] and is designed to serve as a support system and communication platform among health coaches, participants with disabilities and their caregivers, and health care providers. AI4DM consists of (1) a telecoaching dashboard, (2) a participant-specific mHealth app, (3) a caregiver-specific mHealth app, and (4) a conversational agent, Amazon Echo (Amazon).

At the center of AI4DM, there is an AI-embedded coaching dashboard. This dashboard will assist health coaches in offering consistent and personalized health coaching for participants. The AI engine will be connected to a commercial ingredient and recipe database and use a food recommendation engine to offer personalized nutrition tips that take into account a given participant’s recommended diet, the participants’ and caregivers’ cuisine preferences, time and food availability, financial limitations, cooking skills (accessibility), and other preferences and limitations. The AI engine will also feature a rule-based expert system to offer personalized PA recommendations based on the participants’ physical conditions, environmental conditions, and preferences. All data generated by the AI engine will be delivered to the participants and their caregivers after approval and tailoring from health coaches. The delivery of information to participants and caregivers will be enabled through multiple channels—smartphones (mHealth apps), conversational agents (eg, Amazon Echo), and telecoaching phone calls—thereby enhancing accessibility to the information. The design and educational content of mHealth apps will be tailored and inclusive of people with all forms of disabilities. Collectively, these AI-assisted and user-centered design features will reduce the time and effort required by health coaches and promote sustained use of the intervention by participants, eventually enabling self-management.

The primary aim of AI4DM is to evaluate the feasibility (ie, process, resource, management, and scientific feasibility), acceptability (based on surveys and interviews with participants, their caregivers, and health coaches), and preliminary efficacy of AI4DM in adults with type 2 DM (T2DM) and permanent impaired mobility. We will use a randomized controlled pilot study design in which 90 adults with T2DM and permanent impaired mobility will be randomized to the AI4DM intervention arm or an attention control arm. The entire recruitment will be conducted nationally, on the web, through the National Center on Health, Physical Activity, and Disability website, associated social media, and ResearchMatch website. The inclusion criteria were as follows: (1) diagnosis of T2DM; (2) glycated hemoglobin ≥8%; (3) 18 to 65 years of age; (4) living with a permanent physical disability such as a spinal cord injury, spina bifida, multiple sclerosis, or stroke; (5) can speak and read English; and (6) availability of a smartphone. This study was approved by the institutional review board, and all screened participants provided informed consent before enrollment.

For the intervention, each participant will be randomly assigned to one of two treatment conditions: (1) AI4DM intervention with telecoaching support and (2) attention control. We planned an active (coach contact) intervention period of 24 weeks (6 months) with 18 coaching calls during that period, followed by a passive period of 24 weeks (6 months) with only technology access to see if self-management behaviors were sustained. Table 1 presents the study design. For further information on the methods for the parent study, see the research protocol paper [19].

**Table 1:** The research design.

Group	Enrollment; weeks 1-2	Pretest; weeks 2-3	AI4DM^a^ intervention			Posttest; weeks 52-53
			Weeks 4-15^b^	Weeks 16-27^b^	Weeks 28-51	
Intervention	✓^c^	✓	Weekly calls and technology access	Biweekly calls and technology access	Only technology access	✓
Attention control	✓	✓	Weekly courtesy calls with no technology access	Biweekly courtesy calls with no technology access	No technology access	✓

^a^AI4DM: artificial intelligence for diabetes mellitus.

^b^Follow-up data collected at the end of weeks 15 and 27 as well (for both arms of the study).

^c^Study activity present.

We have developed methodological strategies to monitor and enhance fidelity based on the NIH BCC Treatment Fidelity recommendations, focusing on *study design, provider training, treatment delivery, treatment receipt, and enactment of treatment skills* [14]. Monitoring intervention fidelity is essential to ensure the internal and external validity of the intervention.

### Study Design

#### Overview

On the basis of the Behavior Change Consortium treatment fidelity recommendations, study designs must ensure that (1) the procedures and implementation are congruent with the presented theory and clinical practices, (2) participants receive the equal *dose* of the treatments, and (3) all procedures would address possible setbacks in implementation. Textbox 1 presents the fidelity of study design and monitoring plan for AI4DM.

**Fidelity of study design and monitoring plan.**

**Goal**

- Intervention will be congruent with presented theory and practice
- Equal treatment dose will be given within and across conditions
- Addressing implementation setbacks

**Description from the National Institutes of Health Behavior Change Consortium**

- Operationalize treatment to optimally reflect theoretical roots; precisely define variables most relevant to “active ingredients” of the intervention
- Ensure equal treatment “dose” (measured by number, frequency, and length of contact) is adequately described and is the same for each subject within and across treatment conditions
- Address possible setbacks in implementation (eg, treatment providers dropping out)

**Fidelity monitoring plan for artificial intelligence for diabetes mellitus**

- Monthly review of coaching call checklist and call logs
- Quarterly review of randomly selected coaching calls
- Quarterly review of content resource bank
- Team meetings to discuss participant progress and protocol adherence
- Review of telecoaching platform and its event log
- Review of adverse event log

#### Ensure That the Procedures and Implementation Are Congruent With the Presented Theory and Clinical Practices

AI4DM is grounded in the SCT view that self-management is not under the complete control of an individual but rather occurs in the context of a larger social environment involving the individual [18]. Thus, AI4DM systematically focuses on improving social support (friend and family support for PA participation), self-efficacy (confidence in one’s ability to be physically active in various situations), outcome expectancies for PA, and self-regulation (exercise goal setting) to understand how the intervention is effective will be systematically targeted through both the features of the app and the content used for in the app and telecoaching. The participant’s app dashboard will focus on the actions the participant needs to take, such as completing blood glucose logging and nutrition logging, whereas the content will be grounded in the SCT [18].

#### Ensure That Participants Receive an Equal Dose of the Treatments

The attention control group will be used to provide an untreated comparison for the AI4DM intervention group and will receive phone calls from the telecoach at the same frequency as the intervention group. These *courtesy calls* are devised to ensure that both groups received the same amount of attention from the telecoach, thereby not influencing or inflating the effect size for future research. Thus, both groups will receive one weekly call for 12 weeks and one biweekly call for the next week. The AI4DM intervention and attention control groups will be exposed to the same data collection measures and protocols.

#### Ensure That All Procedures Would Address Possible Setbacks in Implementation

We will collect evidence on reporting and handling constraints of adverse events, serious adverse events, and clinical emergencies, as well as participant experiences, burdens, and compliance during the assessment experience and intervention. Assessment experience will be documented through participant self-reports of time to complete the baseline intake. Intervention experience will be documented by having participants self-report parameters of coaching sessions in participant logs and through an evaluation of staff logs.

### Provider Training

The project team of the study has the appropriate experience and training needed for the successful completion of all the proposed activities. We have assembled a multidisciplinary team of academic, technical, and programmatic professionals that are uniquely positioned with the necessary technical, disability, diabetes, PA, nutrition, behavior change, content development, and coaching-related skills and knowledge. During the intervention, health coaches will be formally trained in a PA-, nutrition-, or nursing-related health profession and pay special attention to regular blood sugar monitoring, setting nutrition goals, and PA goals in weekly calls (up to 60 minutes). The dashboard in the developed platform will assist health coaches in offering consistent and personalized health coaching for participants. The Behavior Change Consortium recommends four strategies to monitor and improve provider training: (1) standardize training, (2) ensure provider skill acquisition, (3) minimize *drift* in provider skills, and (4) accommodate provider differences. Textbox 2 outlines the fidelity of the provider or health coach training strategies and the monitoring plan for AI4DM.

**Fidelity of health coach training strategies.**

**Goal**

- Standardized training
- Ensure provider skill acquisition
- Minimize “drift” in provider skills
- Accommodate provider differences

**Description from the National Institutes of Health Behavior Change Consortium**

- Training must be conducted similarly for all providers
- Train providers to well-defined performance criteria
- Ensure provider skills do not decay over time
- Ensure adequate level of training in providers of different skills level, experience, or professional background

**Strategies used in artificial intelligence for diabetes mellitus**

- The coaches will receive American Council on Exercise health coaching training
- Review of coaching calls checklists and call logs
- Recording coaching calls
- Quarterly review of random selection of calls
- Team meetings to discuss participant progress and protocol adherence

### Delivery of Treatment

#### Overview

Another essential strategy provided by NIH BCC is to ensure that treatment or intervention is delivered as planned. As recommended by NIH BCC, we designed our protocol to address four major goals for fidelity of treatment delivery: *control for provider differences,* *reduction of differences within treatment groups,* *ensure adherence to treatment protocols,* and *minimize contamination between conditions* [12,14] (see Textbox 3 for the fidelity of intervention delivery strategies of AI4DM).

**Fidelity of intervention delivery strategies.**

**Goal**

- Control for provider differences
- Reduce differences within treatment
- Ensure adherence to treatment protocol
- Minimize contamination between treatments

**Description from the National Institutes of Health Behavior Change Consortium**

- Monitor and control for subjects’ perceptions of nonspecific treatment effects (eg, warmth and credibility) across conditions
- Ensure that providers in the same condition are delivering the same intervention
- Ensure that the treatments are being delivered in the way in which they were conceived with regard to content and dose
- Minimize contamination

**Strategies used in artificial intelligence for diabetes mellitus**

- Coaching call checklists and call logs
- Random audit of audio recording of coaching calls
- Review of randomly selected coaching sessions
- Having different supervisors to evaluate the content and coaching sessions
- Team meetings to discuss participant progress and protocol adherence
- Review of telecoaching platform and its event log

#### Control for Provider Differences

To deliver the intervention protocol effectively, we plan to incorporate various strategies, including reporting and monitoring participants’ and health coaches’ complaints, having health coaches working with participants in both arms, audiotaping coaching sessions, and auditing and monitoring of random selection of coaching calls by an expert to evaluate the sessions. Although the platform and REDCap (Research Electronic Data Capture) [20] will provide data regarding adherence and retention, an exit survey will be conducted to provide information about the satisfaction and perceptions of the participants. On the basis of the exit survey results and the data on REDCap and platform, 15 participants will be chosen to interview to ask the reason for their low-high adherence, low-high satisfaction, or dropping out.

#### Reduce Differences Within Treatment Groups

AI4DM, as a telecoaching platform, uses an artificial engine–embedded telecoaching dashboard to promote the fidelity of coaching sessions and reduce the workload required to personalize telecoaching. The dashboard will assist the coach in effectively preparing for and conducting calls and acting as a proxy for the participants’ self-management activities if needed. While using a scripted intervention protocol and detailed coaching manuals to reduce human error, we will also use standard materials and resources scheduled for each week based on the PRIDE (Partnership to Improve Diabetes Education) toolkit that has 30 comprehensive interactive education modules [21].

All procedures for delivering treatment goals will be standardized according to the group assignment to reduce the differences within treatment groups. For the coaching sessions, health coaches will focus on four domains: healthy eating, exercise, glucose monitoring, and medication adherence. Although the attention control arm will receive phone calls from the telecoach at the same frequency as the intervention group, these *courtesy calls* will only focus on the general well-being of the participants. Besides the coaching sessions, the platform will involve an abundance of videos, graphical and textual content, and technical features to deliver the intervention effectively. We will maximize the use of short video clips and infographics, paired with appropriate alternate access strategies. Educational content will be provided through the app and reinforced through telecoaching sessions and conversational agents (Amazon Echo). Conducting weekly team meetings to discuss participant progress and protocol adherence and having different supervisors evaluate the content and coaching sessions will also help ensure adequate treatment delivery.

#### Ensure Adherence to Treatment Protocols

In addition to the quarterly audio recording of coaching calls, coaching call checklists and coaching call logs will be used to improve adherence to the intervention protocols. Call logs will include information regarding the calls made, attempted, missed, and voicemails. Scheduled calls and summary notes will also be recorded in these logs. Coaching call checklists are intended to remind health coaches to prepare for scheduled content delivery. The call logs and coaching checklists will be audited monthly. Moreover, a detailed scripted missed call protocol will be provided for health coaches to follow.

#### Minimize Contamination Between Conditions

Upon randomization, participants will be assigned to either the attention control arm or the intervention arm. The coaches will be provided with a scripted protocol for both groups. The coaches will be given standardized training and supplied with the PRIDE manual. The review of recorded phone calls and the trackable progress of the participants on the platform will act as a monitoring step to detect contamination.

### Receipt of Treatment

The success of an intervention depends on its clarity and applicability. Receipt of treatment strategy focuses on the participant’s ability to understand the given content and perform the related behavioral activities during the intervention. The structure of AI4DM was created according to the three goals that NIH BCC suggests: (1) ensure participant comprehension, (2) ensure participant ability to use cognitive skills, and (3) ensure participants’ ability to perform behavioral skills [14].

AI4DM is developed for people with disabilities who are more prone to experiencing low health literacy (LHL) [22,23]. Similarly, older adults, people with less than a high school degree, racial and ethnic minorities, people with low-income levels, and nonnative speakers of English are most likely to experience LHL [24]. Using plain language is necessary to improve health literacy and make written and oral information easier to understand [24]. Improving health literacy among participants with LHL would be beneficial for the education, treatment, and management of chronic diseases. Therefore, all contents of the AI4DM were designed to target the reading level of fifth grade or below (Textbox 4).

In addition, participants will be counseled using techniques drawn from cognitive behavioral therapy [25]. The cognitive behavioral therapy materials in AI4DM will help the participant and caregiver to focus on motivation for change, increasing healthy behaviors (including appropriate diet and exercise), teaching positive coping strategies, and stress management skills. They will also guide the telecoach in using motivational interviewing techniques to introduce the program, assess how ready and willing the participants are to change, and help finalize relevant goals associated with the psychosocial aspects of behavior change (Textbox 4).

**Fidelity of receipt of treatment strategies.**

**Goal**

- Ensure participant comprehension
- Ensure participant ability to use cognitive skills
- Ensure participant ability to perform behavioral skills

**Description from the National Institutes of Health Behavior Change Consortium**

- Ensure that participants understand the information provided by the intervention
- Make sure that participants are able to use the cognitive skills taught in the intervention
- Make sure that participants are able to use behavioral skills taught in the intervention

**Strategies used in artificial intelligence for diabetes mellitus**

- Target reading level of fifth grade or below for the content
- Review of recorded coaching sessions
- Review of participant food, physical activity, medication, and glucose journals
- Review of participant log-ins and time spent on platform
- Team meetings to discuss participant progress and protocol adherence

### Enactment of Treatment Skills

According to NIH BCC, enactment of treatment skills is one of the most challenging aspects of intervention fidelity. It involves applying the proposed theories and protocols to *real-world* settings. To ensure participants’ use of cognitive and behavioral skills, the content for the proposed program will be adapted from a variety of sources. The Diabetes Literacy and Numeracy Education Toolkit [26] and the PRIDE toolkit [21] will form the underlying basis for creating the content. The majority of our efforts will focus on packing the content in a highly accessible yet engaging format. We will maximize the use of short video clips and infographics, paired with appropriate alternate access strategies. Educational content will be provided through mHealth apps and reinforced through telecoaching sessions and conversational agents (Amazon Echo).

Participants in the intervention arm would also receive a wireless blood glucose monitor and a Fitbit Flex (Google LLC), which are used for intervention delivery and not for outcome measurement. This program involves an abundance of videos, graphical and textual content, and technical features such as AI-assisted dietary planning and tracking, sensor-based PA tracking, and context-aware recommendations to help participants understand and adopt the related cognitive and behavioral skills. Having access to digital content (texts and videos) and one-to-one interaction with a health coach is expected to improve the cognitive and behavioral performance of the participants. Textbox 5 presents the strategies that will be used in the AI4DM for the enactment of treatment skills.

**Fidelity of enactment of treatment strategies.**

**Goal**

- Ensure participant use of cognitive skills
- Ensure participant use of behavioral skills

**Description from the National Institutes of Health Behavior Change Consortium**

- Ensure that participants actually use the cognitive skills provided in the intervention in appropriate life settings
- Ensure that participants actually use the behavioral skills provided in the intervention in appropriate life settings

**Strategies used in artificial intelligence for diabetes mellitus**

- Review of participant food, physical activity, medication, and glucose journals
- Review of participant log-ins and time spent on platform
- Team meetings to discuss participant progress and protocol adherence
- Review of coaching notes

## Results

Enrollment for AI4DM began in August 2021 and will end in August 2022. The results of this study will be reported in late 2022.

## Discussion

Treatment fidelity refers to strategies for monitoring and enhancing the consistency and accuracy of an intervention. Reporting treatment fidelity is an essential component to ensure that the intervention is implemented as planned [14,27]. A behavior change intervention must be consistently administered throughout the trial to obtain valid conclusions from the study. Maintaining high intervention fidelity would provide valuable information on the feasibility of the intervention in real-world settings. It can help to minimize experimenter bias and the reactivity of observations by ensuring that any changes observed during an intervention reflect the subject’s behavior only [28,29]. This paper aims to present an overview of the intervention protocol for an ongoing randomized controlled trial that aims to develop and assess a telehealth platform, AI4DM, paired with inclusive diabetes self-management content. AI4DM is grounded in the SCT [18] and will be designed to serve as a support system and communication platform between health coaches, participants with disabilities, and health care providers.

This study applied treatment fidelity strategies and recommendations from NIH BCC, which includes five fidelity domains [14]. We have outlined the strategies for assessing, monitoring, and enhancing our intervention fidelity based on each of these domains. NIH BCC acts as an important and comprehensive resource for providing essential strategies to ensure the internal and external validity of health behavior change studies [30]. AI4DM will be the first web-based diabetes self-management program for people with disabilities that is accessible, inclusive, scalable, and sustainable. Therefore, by reporting the intervention fidelity protocol in accordance with NIH BCC recommendations, this paper provides guidance for other researchers conducting telehealth interventions within behavioral health settings by serving as an example to develop fidelity protocols.

Following the NIH BCC framework, we generated detailed protocols regarding the design, training, delivery, receipt, and enactment components for the management and implementation of our program. One of the challenges that we encountered was using different platforms to manage and monitor the fidelity of the intervention. Comprehensive protocols that use both an AI-assisted telecoaching platform and a data management tool, that is, REDCap, to manage and deliver the intervention. To prevent potential setbacks in the implementation of the intervention and increase the applicability of the procedures, we collaborated with our tech team before finalizing our protocols. Thus, we expected no drastic changes in the fidelity processes during the intervention.

We have observed extensive preparatory and postwork before and after a call through several of our earlier health coaching–related projects. This often involves reading call notes, search recipes, manually emailing reminders, and follow-up messages. Similarly, some studies have pointed out that variations between coaches are a major limitation for health coaching [16,31]. All AI-related features and fidelity-related features also aim to reduce the time required by the coaches, thereby leading to scalability and sustainability of self-management behavior by the participants. However, including telecoaching sessions in the intervention introduces some challenges, such as *therapist drift*, which can lead to lower treatment engagement. In this phenomenon, health coaches may unintentionally or unknowingly shift from the main topic by making small changes to the administration of the intervention [27,32]. In this study, we generated detailed and standardized health coaching protocols and checklists and provided standardized training to prevent potential coaching drifts and maintain intervention integrity. This will help to structure more effective coaching sessions and potentially affect the internal validity of the study. Such standardization can contribute to creating evidence-based coaching sessions that can be incorporated into other behavior change studies.

Although there is always a threat to fidelity assessment that participants can provide desirable responses to the coaches, having a protocol based on NIH BCC criteria will positively affect the study’s internal or external validity, which will eventually affect the outcomes and interpretation of the results. Consistently monitoring fidelity can also provide valuable insights regarding the implementation of the intervention. Therefore, in this study, we outlined all the intervention fidelity strategies and procedures of the AI4DM project to evaluate its feasibility, acceptability, and preliminary efficacy in adults with T2DM and permanent impaired mobility.

## Appendix

Multimedia Appendix 1 Peer-reviewer report from the National Institute on Disability, Independent Living, and Rehabilitation Research.

## Abbreviations

AI: artificial intelligence
AI4DM: artificial intelligence for diabetes mellitus
DM: diabetes mellitus
LHL: low health literacy
mHealth: mobile health
NIH BCC: National Institutes of Health Behavior Change Consortium
PA: physical activity
PRIDE: Partnership to Improve Diabetes Education
REDCap: Research Electronic Data Capture
SCT: social cognitive theory
T2D: type 2 diabetes
T2DM: type 2 diabetes mellitus
